# In Vitro Immune-Enhancement and Anti-Inflammatory Effects of Fatty Acids Extracted from the *Halocynthia aurantium* Gonad on RAW264.7 Macrophages

**DOI:** 10.3390/nu14214510

**Published:** 2022-10-26

**Authors:** Junhyeok Lim, Weerawan Rod-in, Chaiwat Monmai, A-yeong Jang, JeongUn Choi, Woo-Jung Park

**Affiliations:** 1 Department of Wellness-Bio Industry, Gangneung-Wonju National University, Gangneung 25457, Gangwon, Korea; 2 Department of Marine Food Science and Technology, Gangneung-Wonju National University, Gangneung 25457, Gangwon, Korea

**Keywords:** immunomodulation, macrophages, fatty acid, *Halocynthia aurantium*, NF-κB, MAPK

## Abstract

Fatty acids extracted from the *Halocynthia aurantium* gonad (HAGF) were shown to be primarily composed of the highest concentrations of eicosapentaenoic acid (EPA) and docosahexaenoic acid (DHA) at 41% and 17% of total fatty acids, respectively. In the present study, HAGF were examined for their immunostimulant and anti-inflammatory effects on RAW264.7 macrophage cells. HAGF were found to significantly boost nitric oxide (NO) production and increase the levels of inducible nitric oxide synthase (iNOS), cyclooxygenase-2 (COX-2), interleukin-1β (IL-1β), IL-6, and tumor necrosis factor (TNF)-α expression in a dose-dependent manner. Moreover, the phosphorylation of c-Jun *N*-terminal kinase/stress-activated protein kinase (JNK), extracellular signal-regulated kinase (ERK), p38, and nuclear factor κB (NF-κB) p65 was up-regulated by the stimulation of RAW264.7 cells with HAGF. When lipopolysaccharide (LPS)—stimulated the macrophages, they also exhibited anti-inflammatory activity via decreasing NO production and immune-related gene expression, Cluster of differentiation (CD) 86 expression, and protein levels in the NF-κB and mitogen-activated protein kinases (MAPK) signaling pathways. Overall, these results indicate that HAGF exert immune-modulatory effects in macrophages.

## 1. Introduction

Immune response modulation is critical for host health because it can boost the immune system and increase resistance to disease [1]; several immunological functions are performed by macrophages, which play a key role in the innate immune system [2]. The three primary roles of macrophages are to present an antigen, phagocytose, and to modulate immunity by releasing cytokines and growth factors [3]. In response to external stimuli, such as LPS, granulocyte-macrophage colony-stimulating factor (GM-CSF), TNF-α, IL-1β, IL-10, and transforming growth factor (TGF)-β, macrophages activate their defensive mechanisms [4]. In addition to triggering the production of NO and pro-inflammatory cytokines, such as IL-1β, IL-6, and TNF-α, the activation of macrophages also triggers the activation of intracellular immune signals, such as the nuclear factor-κB (NF-κB) and mitogen-activated protein kinase (MAPK) pathways [5].

In general, fatty acids (FAs) are categorized as saturated fatty acids (SFAs), monounsaturated fatty acids (MUFAs), or polyunsaturated fatty acids (PUFAs), depending on the quantity of double bonds within the molecule [6]. These compounds are associated with resistance to infections and the immune system, as well as inflammation and the response to stress [7,8]. The Omega-3 PUFAs that can be converted into lipid mediators are eicosapentaenoic acid (EPA) and docosahexaenoic acid (DHA), which are mainly found in marine sources [9,10,11]. Both DHA and EPA exhibit anti-inflammatory and immunomodulatory effects [12,13,14,15,16]. Studies of marine ascidians have demonstrated that FAs stimulate macrophages to produce NO, prostaglandins, and cytokines, which enhances their cytotoxic and immunomodulatory effects [11,17,18]. They also activate immune signaling pathways, such as MAPK and NF-κB [11,19,20,21,22].

*Halocynthia aurantium*, a member of the Pyuridae family, can often be found in the northern Pacific Ocean, from the Arctic Sea south to Puget Sound [23]. Several biological functions have been studied in this genus, including antibacterial, anti-proliferation, anti-diabetic, antioxidant, anti-inflammatory, and immunomodulatory activities [17,24,25,26]. In macrophages, the carotenoids derived from *H. roretzi* were isolated and they showed anti-inflammatory effects on LPS-stimulated RAW264.7 cells [27], whereas polysaccharides from the tunic of *H. roretzi* also had immunomodulatory effects in RAW264.7 cells [28].

In addition, FAs isolated from the body walls and tunics of *H. aurantium* have also been shown to exhibit immunomodulation in RAW264.7 cells, which is regulated by NO and PGE_2_ [11,17]. It also showed a variation in gene expression through the activation of the MAPK and NF-κB signaling pathways in RAW264.7 cells. However, none of these studies have explored the effect of fatty acids extracted from the *Halocynthia aurantium* gonad (HAGF) on immune cells. Therefore, this study is the first to evaluate the immunomodulatory effects and the underlying mechanisms of HAGF in RAW264.7 macrophages.

## 2. Materials and Methods

### 2.1. Samples

*H. aurantium* was obtained from the Jumunjin fish market on the east coast near Gangwon Province, Korea. The gonads were separated from the whole body and collected. The samples were immediately stored at a temperature of −20 °C, after dissection of the body.

### 2.2. Fatty Acid Extraction and Fatty Acid Profile Analysis

Garces and Mancha’s method for extracting HAGF was followed [29]. Fatty acid methyl esters (FAMEs) were synthesized by performing hydrolyzation, extraction, and methylation [29,30]. Fatty acid analyses and cell experiments were carried out using FAMEs. Gas chromatography (GC) with flame ionization detection (Perkin Elmer, Waltham, MA, USA) and the HP-5 GC capillary column (30 m × 0.32 mm internal diameter (i.d.), 0.25 μm film thickness, Agilent Technologies, Santa Clara, CA, USA) were used to examine the FA profiles.

### 2.3. Cell Culture

A murine macrophage RAW264.7 cell line was provided by the Korean Cell Line Bank (Korean Cell Line Research Foundation, Seoul, Korea). The cells were grown in a Roswell Park Memorial Institute (RPMI) 1640 medium with 10% fetal bovine serum (FBS) and 1% penicillin/streptomycin supplementation at 37 °C, in a humidified incubator with 5% CO_2._

### 2.4. Macrophage Cell Viability Assay

RAW264.7 cells were seeded into a 96-well plate at a density of 1 × 10^6^ cells/mL. Different concentrations of HAGF, at 0.5%, 1.0%, 1.5%, and 2.0%, were administered to RAW264.7 cells, and stimulation was performed with or without lipopolysaccharides (LPS) at 1 µg/mL. After culturing the cells for another 24 h, the supernatants were removed to test the cytotoxicity of FAs. The Water Soluble Tetrazolium Salt (WST) solution, in an EZ-Cytox Cell Viability Assay Kit (Daeil Lab Service, Seoul, Korea), was added to the cells and incubated at 37 °C for 1 h. After incubation, the absorbance of the solution was measured at 450 nm.

### 2.5. Nitric Oxide (NO) Production Assay

The cells were treated with HAGF at various doses (0.5%, 1.0%, 1.5%, and 2.0%), and stimulation was performed with or without LPS. Griess reagents (Promega, Madison, WI, USA) were used to assess the immune-regulatory ability of RAW264.7 cells for NO generation of FAs [31]. Briefly, 100 μL of the cultured supernatants was combined with 50 μL of Griess reagent A (1% sulfanilamide in 5% phosphoric acid) and 50 μL of Griess reagent B (0.1% *N*-1-napthylethylenediamine dihydrochloride in water). After incubation, the absorbance of the solution was measured at 540 nm.

### 2.6. RNA Isolation and Real-Time PCR

TRI reagent^®^ (Molecular Research Center, Inc., Cincinnati, OH, USA) was used to isolate the total RNA from the cells. The lysate was homogenized with 200 µL of chloroform after being transferred to a 1.5 mL microtube. After that, centrifugation was performed at 13,000 rpm at 4 °C for 10 min. In a new 1.5 mL microtube, the supernatant and isopropanol were added and incubated at 4 °C for 30 min. The pellet was obtained after 10 min of centrifugation at 13,000 rpm at 4 °C. Before being dissolved in nuclease-free water and maintained at −20 °C, the total RNA was washed three times with 70% ethanol. The cDNA was produced using the High-Capacity cDNA Reverse Transcription Kit (Applied Biosystems, Waltham, MA, USA), after the RNA was extracted.

The expression of immune genes was measured using amplification reactions, including 5 ng of cDNA, SYBR Premix EX Taq II (Takara Bio Inc., Kusatsu, Japan), and the specific primer sets of IL-1β, IL-6, TNF-α, iNOS, COX-2, and β-actin [32]. On a QuantStudio™ 3 FlexReal-Time PCR System (Applied Biosystems, Waltham, MA, USA), the experiment was carried out in triplicate and analyzed.

### 2.7. Western Blot Assay

A radio-immunoprecipitation assay buffer (Tech & Innovation, Zhangjiakou, China), with a 0.5 mM EDTA solution and 0.1% protease and phosphatase inhibitor cocktails (Thermo Fisher Scientific, Waltham, MA, USA), was used to separate the protein from treated FA cells. Pierce™ BCA protein analysis (Thermo Fisher Scientific, Waltham, MA, USA) was used to measure the isolated proteins. SDS-polyacrylamide gel was employed to separate the same amount of proteins (30 µg), which were then transferred to polyvinylidene fluoride membranes (Merck, Kenilworth, NJ, USA) in each treatment. The membrane was blocked with 5% skim milk in TBST buffer (1 × Tris-buffer saline (TBS) and 0.1% Tween 20) for 1 h. Based on the previous reports [33], protein levels were estimated using Western blot analysis. Initially, incubation with primary antibodies against phospho-p44/42 MAPK (Erk1/2) (Thr202/Tyr204) (dilution 1:2000; #9101; Cell Signaling Technology, Danvers, MA, USA), phospho-SAPK/JNK (Thr183/Tyr185) (dilution 1:2000; #9251; Cell Signaling Technology, Danvers, MA, USA), phospho-p38 MAPK (Thr180/Tyr182) (dilution 1:2000; #9211; Cell Signaling Technology, Danvers, MA, USA), phospho-NF-κB p65 (Ser536) (dilution 1:2000; #3033; Cell Signaling Technology, Danvers, MA, USA), and α-tubulin (dilution 1:2000; ab15246; Abcam, Cambridge, UK) was performed on the membrane overnight at 4 °C. Following that, a secondary antibody, goat anti-rabbit IgG (H+L)-horseradish peroxidase (HRP) (dilution 1:2000; SA006-500; GenDEPOT, Katy, TX, USA), was then incubated on the membrane for 1 h at room temperature. Pierce^®^ ECL Plus Western Blotting Substrate (Thermo Scientific, Waltham, MA, USA) and ImageLab software (version 4.1, Bio-Rad, Hercules, CA, USA)) were used to identify the protein signal.

### 2.8. Phagocytic Uptake of Macrophages

The quantity of surface molecule expression on macrophages was analyzed, as previously described [34]. The cells were activated with HAGF (0.5–2.0%) and LPS. The cells were collected and washed in a cold FACS buffer after being incubated for 24 h. The cells were coated with fluorescein isothiocyanate (FITC)-dextran (Sigma-Aldrich, St. Louis, MO, USA), for 30 min. After incubation, the cells were washed with FACS buffer. The cells were analyzed using a CytoFLEX Flow Cytometer (Beckman Coulter, Inc., Brea, CA, USA) after being added with 300 μL of FACS buffer.

### 2.9. Analysis of Expression of Surface Molecules

The quantity of cell surface molecule expression on macrophages was analyzed as previously described [34]. The cells were activated with HAGF and LPS. The cells were collected and washed in cold FACS buffer after being incubated for 24 h. Purified rat IgG (Invitrogen, USA) was blocked to the cells for 10 min. The cells were coated with CD40-PE and CD86-APC (Invitrogen, Carlsbad, CA, USA) antibodies, as well as their isotype controls, for an additional 10 min. After incubation, the cells were washed with FACS buffer. The cells were analyzed with a CytoFLEX Flow Cytometer (Beckman Coulter, Inc., Brea, CA, USA) after being added with 300 μL of FACS buffer.

### 2.10. Statistical Analysis

The data are displayed as means and standard deviations (SD). In order to determine the differences between treatments, Tukey’s pairwise comparisons and one-way ANOVA were used. At *p* < 0.05, statistical significance was declared.

## 3. Results

### 3.1. Fatty Acid Profiles of HAGF

**Figure 1:**
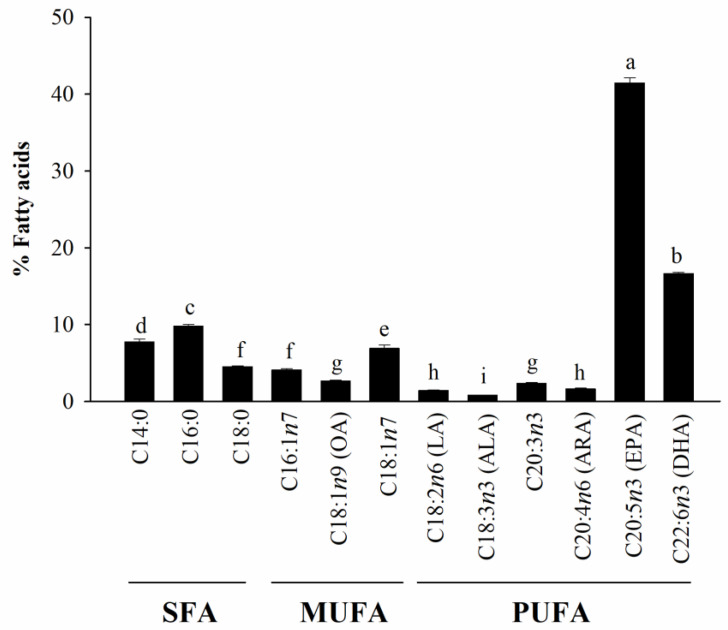
Fatty acid compositions of HAGF. Values are shown as means ± SD (*n* = 5). Different letters (a–i) are used to reflect statistical differences at *p* < 0.05.

The compositions of important FAs of HAGF are shown in Figure 1. The FA compositions in this sample were as follows: SFAs with 22.00 ± 0.48%, MUFAs with 13.63 ± 0.28%, and PUFAs with 64.37 ± 0.75%. Myristic acid (C14:0), palmitic acid (C16:0), and stearic acid (C18:0) were the predominant SFAs, whereas C18:1*n*7 was the main MUFA instead of 18:1*n*9. The PUFAs consisted of linoleic acid (18:2*n*-6; LA), linolenic acid (18:3*n*-3; ALA), eicosatrienoic acid (ETA; C20:3*n*-3), arachidonic acid (ARA; C20:4*n*6), EPA (C20:5*n*-3), and DHA (C22:6*n*-3). Among the PUFAs, EPA and DHA showed the highest content, with 41.50 ± 0.62% and 16.65 ± 0.18% of the total FAs, respectively.

### 3.2. Effects of HAGF on Cytotoxicity and NO Production in RAW264.7 Cells

**Figure 2:**
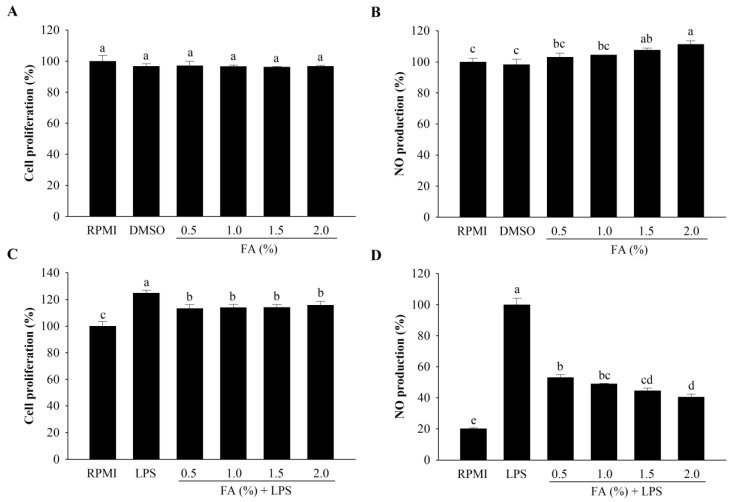
The effect of HAGF on cell proliferation and production of NO. (**A**) RAW264.7 cell proliferation. (**B**) NO production in RAW264.7 cells. (**C**) LPS-stimulated proliferation. (**D**) LPS-stimulated NO production. Values are shown as means ± SD (*n* = 3). Different letters (a–e) are used to reflect statistical differences at *p* < 0.05. FA = fatty acid.

RAW264.7 cells were tested using the EZ-Cytox Cell Viability Assay for their viability after treatment with HAGF. The HAGF (0.5–2.0%) showed minimal toxicity but it was not statistically different (*p* < 0.05) in macrophage cells (Figure 2A). However, a significant increase in proliferation was observed in LPS-stimulated RAW264.7 cells after treatment with HAGF (Figure 2C). In addition, to assess the immune-regulatory effects of HAGF, the production of NO was investigated. As shown in Figure 2B, the immune-enhanced effects of HAGF boosted NO production in a dose-dependent manner, according to the 0.5–2.0% concentrations of FAs used. Conversely, LPS-induced NO production was considerably reduced by the HAGF (0.5–2.0%) (Figure 2D).

### 3.3. Effects of HAGF on the Expression of Immune-Related Genes

**Figure 3:**
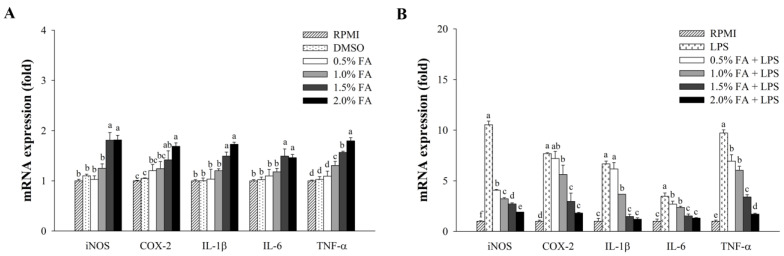
The effect of HAGF on gene expression associated with immune responses in RAW264.7 cells. (**A**) The mRNA expression without LPS stimulation. (**B**) LPS-stimulated mRNA expression. Values are shown as means ± SD (*n* = 3). Different letters (a–f) are used to reflect statistical differences at *p* < 0.05. FA = fatty acid.

Figure 3 shows the immunomodulatory effects of HAGF, which were mediated by activating immune-related genes. As shown in Figure 3A, concentration-dependent increases in the mRNA expression of IL-1β, IL-6, TNF-α, iNOS, and COX-2 were observed after treatment with HAGF. As shown in Figure 3B, the cells were treated over a range of concentrations (0.5%, 1.0%, 1.5%, and 2.0%), without and with LPS stimulation. Our study revealed that the mRNA expression was considerably higher in LPS-treated cells than in control cells (RPMI). HAGF significantly down-regulated the mRNA expression in a dose-dependent manner.

### 3.4. Effects of HAGF on the Activation of NF-κB and MAPK Signaling Pathways

**Figure 4:**
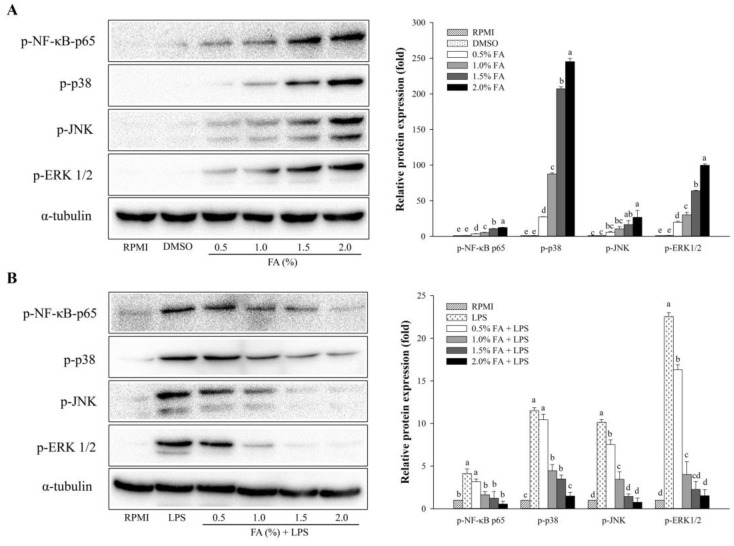
The effect of HAGF on NF-κB and MAPK activation. (**A**) RAW264.7 cell protein expression. (**B**) Protein expression in RAW264.7 cells stimulated with LPS. Values are shown as means ± SD (*n* = 3). Different letters (a–e) are used to reflect statistical differences at *p* < 0.05. FA = fatty acid.

As shown in Figure 4, Western blotting was used to assess the expression levels of NF-κB- and MAPK-related proteins. A dose-dependent effect of HAGF was observed for NF-κB p-65 phosphorylation in the NF-κB pathway (Figure 4A). As part of the MAPK pathway, HAGF also dose-dependently increased the phosphorylation of JNK, p38, and ERK1/2 (Figure 4A). Unlike in Figure 4A, the treatment with LPS, shown in Figure 4B, raised the protein expression of phosphorylated NF-κB p65 subunits, while 0.5–2.0% of HAGF markedly inhibited the LPS-induced NF-κB phosphorylation. Furthermore, LPS-induced phosphorylation of ERK1/2, JNK, and p38 in cells was dose-dependently suppressed by HAGF (Figure 4B).

### 3.5. Effects of HAGF on the Phagocytosis of Macrophages

**Figure 5:**
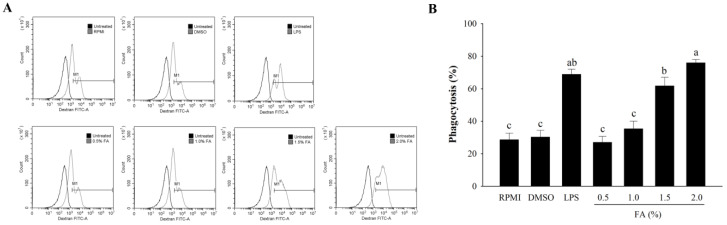
The effect of HAGF on the phagocytosis of macrophages. (**A**) Fluorescence histograms of FITC–dextran uptake. (**B**) The percentage of phagocytic activity. Values are shown as means ± SD (*n* = 3). Different letters (a–c) are used to reflect statistical differences at *p* < 0.05. M1 = An immune stimulation activity-related macrophage phenotype. FA = fatty acid.

An analysis of macrophage phagocytosis by HAGF was performed using FITC-dextran. As shown in Figure 5, the phagocytic activity was strongly elevated by LPS, as compared with RPMI as the negative control. Treatment with DMSO, as a model control, did not increase the phagocytic activity. HAGF (0.5–2.0%) reinforced the phagocytosis effects on RAW264.7 cells in a dose-dependent manner. However, exposure to high concentrations of HAGF (1.5–2.0%) greatly increased the phagocytic activity.

### 3.6. Anti-Inflammatory Effects of HAGF on Cell Surface Molecule Expression

**Figure 6:**
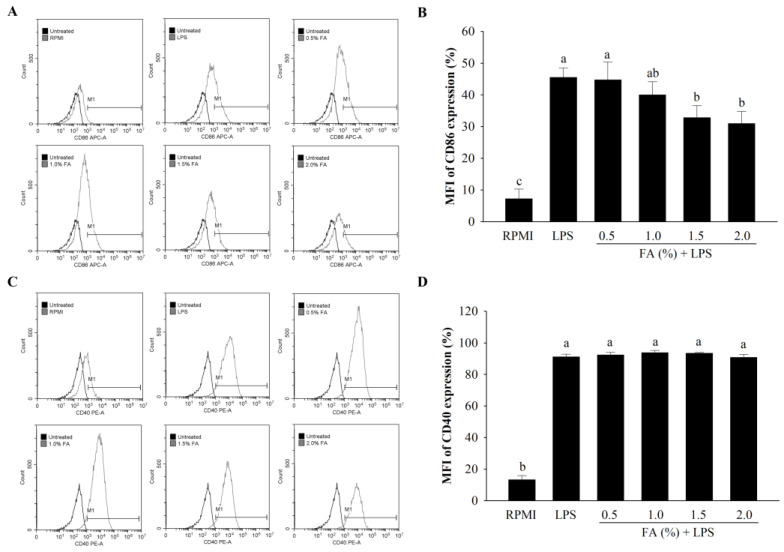
The effect of HAGF on the cell surface molecule expression. (**A**) CD86 expression fluorescence histograms. (**B**) Mean fluorescence intensity (MFI) of CD86 expression. (**C**) CD40 expression fluorescence histograms. (**D**) MFI of CD40 expression. Values are shown as means ± SD (*n* = 3). Different letters (a–c) are used to reflect statistical differences at *p* < 0.05. M1 = An immune stimulation activity-related macrophage phenotype. FA = fatty acid.

The expression levels of cell surface molecules, such as CD40 and CD86, were determined by performing an FACS analysis in LPS-stimulated cells. A concentration-dependent inhibition of CD86 expression on the cell surface was observed with HAGF (Figure 6A,B). Interestingly, CD40 expression did not change after treatment with samples (Figure 6C,D).

## 4. Discussion

In the present study, FAs were extracted from *H. aurantium* gonads. These FAs were investigated in terms of their composition, and immune-enhancing and anti-inflammatory activities in RAW264.7 cells. Several studies have shown that FAs modulate immune function [11,14,15,16].

The FA profiles of HAGF were analyzed using gas chromatography. Our results demonstrated that myristic acid, palmitic acid, stearic acid, C18:1*n*7, EPA, and DHA are the major components of HAGF. In *H. aurantium*, the main FAs of gonads were similar to those of the body wall and tunic [11,17], but both organs contained a different number of FAs. In the tunic of *H. aurantium*, levels of SFAs (53.17%) were higher than PUFAs (34.65%), whereas there were more PUFAs than SFAs in the gonads.

Macrophages are a type of immune cell that release cytotoxic and inflammatory chemicals, such as NO, and secrete cytokines in response to pathogens external to the body [3]. NO is a critical biomarker for inflammatory mediators and can increase the release of specific hormones involved in immunological regulation in the immune system [35]. Our results showed that HAGF at 0.5–2.0% had no cytotoxic effect, and the production of NO stimulated by non-LPS and LPS conditions was not associated with cell cytotoxicity. *H. aurantium* tunic FAs (0.5–2.0%) have immune-enhancing and anti-inflammatory activities, in regulating NO and PGE_2_ production as well as the expression of iNOS, IL-1β, IL-6, COX-2, and TNF-α in macrophages [11]. *Alysia fasciata* and *A. punctate* FAs have shown anti-inflammatory properties by inhibiting LPS-stimulated NO production [18]. Similarly, our results revealed that HAGF increased the expression of iNOS, COX-2, IL-1β, IL-6, and TNF-α in RAW264.7 cells. In contrast, they also decreased the LPS-induced gene expression.

In addition, FAs activated the regulation of NF-κB and MAPK pathways in macrophages, which are involved in inflammatory responses [20,22,36]. NF-κB, as a transcription factor, is essential for the survival, activation, and differentiation of innate immune cells and inflammatory T cells [37]. MAPKs, including extracellular signal-regulated kinases (ERKs) and stress-activated protein kinases (SAPKs), such as p38 and c-jun *N*-terminal kinase (JNK), regulate cellular processes, including cell growth, differentiation, and gene expression. MAPKs also contribute to cellular stress and inflammation [38]. The results suggest that HAGF enhanced and suppressed phosphorylated NF-κB p65, ERK1/2, JNK, and p38. In a previous study, the body wall and tunic of *H. aurantium* were used as a source of FAs that showed immune-stimulating and anti-inflammatory properties through activating the NF-κB and MAPK pathways [11,17]. These results suggest that HAGF may exhibit immune-regulatory effects on inflammatory mediators and pro-inflammatory cytokines through NF-κB and MAPK activation in macrophage cells.

Phagocytosis is caused by various receptors, where ligands bind and interact with cells to receive signals [39]. Generally, macrophages have a phagocytic function and are involved in the early stages of immunity and inflammation [40,41]. HAGF were found to enhance phagocytosis activity, and the effects of these FAs at 1.5% and 2.0% were comparable to those of LPS. The results suggest that HAGF could activate macrophages, which corresponds to the findings in previous reports [32,42]. The expression levels of accessory/costimulatory molecules on the surface of macrophages, such as CD40 and CD86, can influence the inflammatory responses [34]. On the cell surface, CD40 and CD86 have a continuous correlation between antigen-presenting cells (APCs) and T cells [43,44]. In our study, CD86 expression was decreased in RAW264.7 cells treated with LPS. CD40 expression did not change significantly. These findings indicate that the immunological control of RAW264.7 cells is mediated by CD86, rather than CD40.

## 5. Conclusions

HAGF, containing high amounts of PUFAs, followed by SFAs and MUFAs, exerted immune-enhancing and anti-inflammatory effects. Stimulation with HAGF increased the production of NO, macrophage cytotoxicity, phagocytosis ability, immune-associated gene expression, and protein expression in RAW264.7 cells. Conversely, HAGF down-regulated NO production, immune-associated gene expression, and CD86 expression, as anti-inflammatory regulations, via the inhibition of LPS-stimulated NF-κB and MAPK activation. Therefore, HAGF may be useful for immune regulation and anti-inflammatory functions.

## Data Availability

Not applicable.
